# Do Life Course Physical Activity Profiles Reduce the Effects of Childhood Exposures on Cognition?

**DOI:** 10.1093/geroni/igab046.104

**Published:** 2021-12-17

**Authors:** Monica Williams-Farrelly, Jacqui Smith

**Affiliations:** University of Michigan, University of Michigan, Michigan, United States

## Abstract

Although physical activity throughout life is one of the most reliable predictors of healthy aging, can less consistent or favorable trajectories also improve cognition trajectories among older adults? Drawing from accumulation theories, we use longitudinal data from the Health and Retirement Study and Life History Mail Survey (N=9,309) to examine the early antecedents of cognitive decline and the extent to which different life course physical activity profiles can slow such a decline. Results from latent class analysis reveal seven distinct profiles: consistently low, consistently high, consistently average (reference), improvers, decliners, midlife motivators, and previously athletic “couch potatoes.” Growth curve modeling analyses show that membership in the consistently high class and midlife motivators were associated with better cognition initially and over time, with no difference between the two classes. Additionally, though poor health and learning problems in childhood were associated with worse initial cognition, physical activity does not mediate the relationship.

